# Renal Cell Carcinoma Presenting with Cutaneous Metastasis: A Case Report 

**DOI:** 10.1155/2010/913734

**Published:** 2010-08-02

**Authors:** Nilufer Onak Kandemir, Figen Barut, Kıvanç Yılmaz, Husnu Tokgoz, Mubin Hosnuter, Sukru Oguz Ozdamar

**Affiliations:** ^1^Department of Pathology, Zonguldak Karaelmas University, Kozlu, 67600 Zonguldak, Turkey; ^2^Department of Urology, Zonguldak Karaelmas University, Kozlu, 67600 Zonguldak, Turkey; ^3^Department of Plastic Reconstructive and Aesthetic Surgery, Zonguldak Karaelmas University, Kozlu, 67600 Zonguldak, Turkey

## Abstract

Renal cell carcinoma is the most common kidney tumor in adults. Cutaneous metastasis is a rare first symptom of the disease. This paper describes the diagnosis of a renal cell carcinoma that was indicated by cutaneous metastasis in the head and neck region, and considers the etiopathogenesis of such cases. A careful skin examination is important to detect cutaneous metastasis associated with renal cell carcinomas. Metastatic skin lesions in the head and neck region must be taken into consideration during a differential diagnosis.

## 1. Introduction

Although visceral organ malignancies infrequently present with cutaneous metastasis, their association is well defined. Cutaneous metastasis develops in 5–10% of high-stage cancer patients, most frequently in association with breast, lung, colon, ovarian, and metastatic malignant melanomas [1]. Although cutaneous metastases rarely develop in cancers of the urogenital system, they can occur in renal cell carcinomas (RCCs). Comprising about 90% of all renal tumors, RCCs are characterized by potential metastatic extension foci in the lymph nodes, lungs, liver, opposite kidney, adrenal glands, brain, and bone [2]. The development of RCC-related cutaneous metastasis in the head and neck region is unusual, given the distance from the anatomical localization and the lymphohematogenous extension pathways of the tumor [3]. 

The RCC case presented here was cutaneous metastasis in the postauricular region. Such a presentation is rare, and its features are discussed in the context of currently available clinical and histopathological knowledge that informed the differential diagnosis.

## 2. Case Presentation

### 2.1. Clinical Features

A 53-year-old male patient presented with swelling behind his right ear that had persisted for more than 1 month and showed a tendency for expansion. Physical examination revealed a painful mass, 3 × 2 × 2 cm in size, that exhibited capillary distinctions and was fixed to adjacent tissues in the right postauricular region. The lesion was clinically diagnosed as a dermoid cyst and was excised completely. The patient had undergone a right nephrectomy due to RCC at a different medical center 3 years previously.

### 2.2. Macroscopic Characteristics

**Figure 1:**
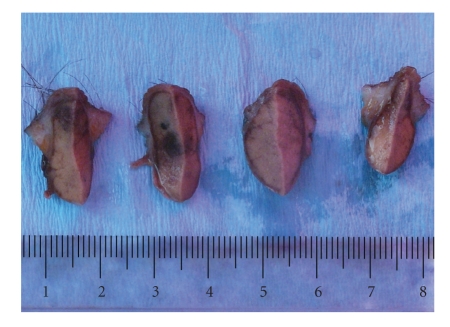
Macrophotograph showing solid tumoral lesion in subepidermal area. A slice of the lesion contained some regions of bleeding.

Gross observation of the biopsy material revealed a bulky erythematous lesion, 3 × 2 × 2 cm in size, on the surface of the skin and underskin tissues. A solid, creamy coffee-colored, nodular lesion was observed in a skin and under skin tissue slice. Smooth boundaries separated the lesion from adjacent tissues. A slice of the lesion contained some regions of bleeding (Figure 1). 

### 2.3. Histopathological Characteristics

**Figure 2:**
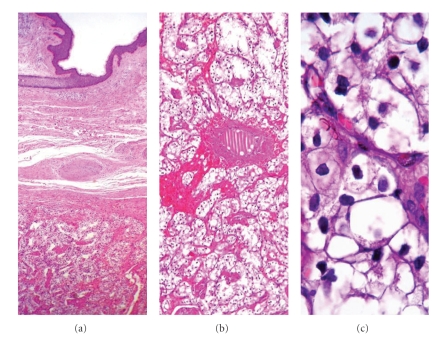
Histological microphotograph showing neoplastic cells with prominent nucleoli and moderate plemorphism beneath the squamous epithelium (H&E; (a) ×100; (b) ×200; (c) ×400).

The H&E-stained slices showed a tumoral lesion that was separated from adjacent structures by thin fibrous tissue. The lesion was localized predominantly in the dermis, beneath hyperkeratotic epidermis, and pressing on subcutaneous fatty tissue (Figure 2(a)). The tumor cells had translucent cytoplasm, prominent cytoplasmic membranes, and round-to-ovoid nuclei (Figure 2(b)). Pleomorphisms in the tumor cell nuclei and eosinophilic nucleoli were apparent (Figure 2(c)). The tumor cells formed acinar structures and solid islets. The fibrovascular stroma showed areas of fresh hemorrhage and hemosiderin-containing histiocytes. The tumoral lesion was separated from the epidermis by thin dermal tissue with congested capillary vessels. The tumor infiltrated the surrounding dermis in focal areas, and no lymph node morphology or vascular tumor embolism was detected. The mitotic activity in the tumor cells was determined to be 3–5/10 high-power fields (HPF). 

### 2.4. Histochemical and Immunohistochemical Characteristics

**Figure 3:**
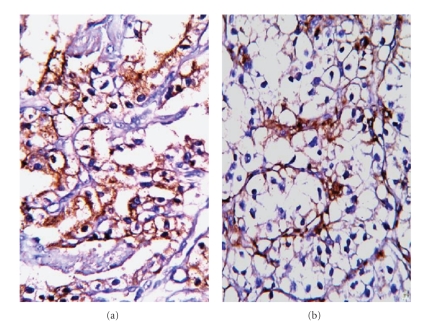
Renal cell carcinoma marker and vimentine immunoreactions in neoplastic tumor cells (ABC-DAB; (a, b) ×200).

**Figure 4:**
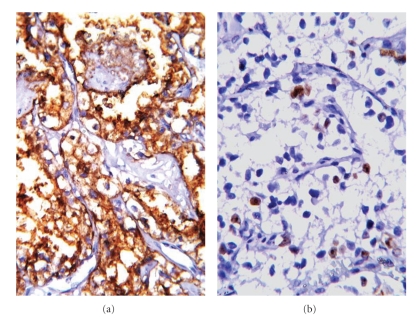
Diffuse positive membranous immunoreaction with antibodies against EMA (a) and focal positive nuclear Ki-67 immunoreaction (b) are observed in the clear cells (ABC-DAB; (a) ×200; (b) ×400).

PAS-stained samples showed intracytoplasmic granules in the neoplastic cells. Mucicarmine staining was uninformative. The tumor cells reacted positively with RCC (Figure 3(a)), vimentin (Figure 3(b)), pan-CK, EMA (Figure 4(a)), and CD10, but no reaction was observed with the other immune indicators. A 5% Ki67 index was determined for the neoplastic cells (Figure 4(b)). These results led to a diagnosis of RCC-related cutaneous metastasis.

## 3. Discussion

RCC comprises 2–3% of all adult malignities. The classical signs of RCC, including hematuria, flank pain, and a palpable abdominal mass, are detected in only 10% of RCC cases. Consequently, most cases are diagnosed during examination for other causes or by the appearance of metastatic lesions [2, 3]. Cutaneous metastasis in RCC is rare; its incidence is only 3.4%. Surprisingly, all reported cutaneous metastasis cases associated with RCC have occurred in males [4, 5]. Our findings support previously reported indications that the potential for cutaneous metastasis in RCC is high in males [2–5]. 

Various mechanisms can cause cutaneous metastases in visceral malignities. The most frequent pathway is the direct invasion of skin tissue covering the malignant mass. Other potential mechanisms include the implantation of neoplastic cells into the skin during surgery or diagnostic procedures, and lymphatic or hematogenous extension [1]. In non-RCC urogenital malignancies, cutaneous metastasis occurs most frequently in the abdominal region [3–5]. In contrast, the head and neck region is most frequently affected in RCC. The rich vascular structure of these tumors facilitates hematogenous extension and the development of distant metastases. The most important hematogenous extension route in RCC involves the right atrium, by way of the vena cava, following the renal vein, and ultimately affecting the lungs. Arteriovenous and systemic shunts are thought to facilitate the tumor's path to the head and neck region by overstepping lung filtration. Tumor-related growth factors, such as parathyroid-related protein and truncated fibronectin growth-promoting substance, may also play an important role in the localization of cutaneous metastasis in this region [6–9]. In the present case, the postauricular metastatic lesion containing vascularly rich tumor tissue suggests that lymphohematogenous extension was likely. This case may also have been characterized by the previously defined pathway in which the primary tumor invades the vertebral veins or Batson's plexus, and tumor cells arrive in the head and neck region by means of intracranial venous pathways.

RCC-related cutaneous metastasis often presents as a solitary, shiny skin lesion that is red-to-purple in color. In some cases, however, the lesions are scattered, plaque-like, or nodular. The rich vascular component of cutaneous metastasis in RCC may cause clinical confusion with hemangiomas, pyogenic granulomas, and Kaposi's sarcoma [3–5, 10]. The morphological appearance of the lesion's surface can also imitate cutaneous cysts, cutaneous horns, lymphomas, or abscesses [11–15]. RCC has been diagnosed through cutaneous metastasis when the primary tumor was too small to be detected or had an involution [8, 16]. In the present case, the postauricular RCC metastasis was clinically diagnosed as skin adnexal tumor. Systemic examination of the patient revealed multiple masses consistent with metastasis in the left adrenal gland, the paraaortic region, and the iliac bone.

The differential diagnosis of metastatic skin lesions may raise important clinical and histopathological issues. The detection of the disease's primary focus and histopathological analysis are important when making decisions about treatment. Cutaneous metastasis in RCC is often characterized by intradermal nodules with a thin dermal tissue space between the epidermis and the tumor tissue [3–5]. Because epidermotropic metastasis is very rare in nonmelanocytic primary tumors, a punch or excisional biopsy is necessary for diagnosis. A shave biopsy is insufficient because dermal involvement may be overlooked. The metastatic lesion in our case was localized primarily in the dermis, and uninvolved dermal tissue was available beneath the epidermis [7–9]. The tumor infiltrated the subcutaneous tissue in patches. Total excision of the lesion allowed complete histomorphological observation and provided sufficient tissue samples for a detailed immunohistochemical survey. 

Most RCC-related cutaneous metastases, including the case presented here, exhibit a histomorphological appearance that is consistent with clear-cell adenocarcinoma [3]. The tumor cells are typically large, with translucent cytoplasm, round-to-oval nuclei, and obvious nucleoli. The tissue cells may form glandular, acinar, or papillary structures. Extravasated erythrocytes are frequently present in the fibrovascular stroma. Cytoplasmic glycogen can be detected with PAS staining [5–9]. The basic histological differential diagnosis is a skin appendage tumor. Benign and malignant sebaceous tumors, eccrine acrospiromas, malignant melanomas with translucent cell appearance, and soft-tissue sarcomas must be ruled out. The differential diagnosis of metastases in the head and neck region must also consider salivary gland tumors and odontogenic tumors. Positive immune reactions of the neoplastic cells with cytokeratin, EMA, vimentin, and CD10 contribute to the histomorphological diagnosis of RCC. Because the lesion discussed here was localized in the head and neck region, the differential diagnosis considered salivary gland tumors, such as mucoepidermoid carcinomas and acinic cell carcinomas, malignant melanomas, and skin appendage tumors. Because the tumor had histomorphological and immunohistochemical features typical of RCC, other lesions were excluded and the case was diagnosed as metastatic RCC [12–19].

The development of cutaneous metastasis in RCC is associated with a poor prognosis. Most patients die within 6 months of cutaneous metastasis detection. Treatment options are thus limited and palliative in nature. Although local excision is an alternative treatment for localized cutaneous metastasis, it often provides little benefit, due to the presence of extensive metastasis. Although radiotherapy has a limited effect on primary renal cell carcinoma, its devascularization of the lesion may be effective in metastatic cases [2]. The case presented here was referred to the medical oncology department as advanced-stage RCC because of extensive metastasis.

Although rare, cutaneous metastasis can be an important manifestation of RCC. Because these lesions imitate other dermatological diseases, a histopathological survey of biopsied samples containing sufficient dermal tissue is essential for diagnosis. RCC must be taken into consideration during the differential diagnosis of tumors with translucent cell morphology localized in the head and neck region.
